# Global prevalence of pre-existing HCV variants resistant to direct-acting antiviral agents (DAAs): mining the GenBank HCV genome data

**DOI:** 10.1038/srep20310

**Published:** 2016-02-04

**Authors:** Zhi-wei Chen, Hu Li, Hong Ren, Peng Hu

**Affiliations:** 1 Department of Infectious Diseases, Institute for Viral Hepatitis, The Key Laboratory of Molecular Biology for Infectious Diseases, Chinese Ministry of Education, The Second Affiliated Hospital of Chongqing Medical University, Chongqing, China

## Abstract

Direct-acting antiviral agents (DAAs) against hepatitis C virus (HCV) proteins open a whole new era for anti-HCV therapy, but DAA resistance associated variants (RAVs) could jeopardize the effectiveness of DAAs. We reported the global prevalence of DAA RAVs using published GenBank data. 58.7% of sequences (854/1455) harbored at least one dominant resistance variant and the highest RAV frequency occurred in Asia (74.1%), followed by Africa (71.9%), America (53.5%) and Europe (51.4%). The highest RAV frequency was observed in genotype (GT) 6 sequences (99%), followed by GT2 (87.9%), GT4 (85.5%), GT1a (56%), GT3 (50.0%) and GT1b (34.3%). Furthermore, 40.0% and 29.6% of sequences were detected RAVs of non-structural (NS) 5A inhibitors and NS3 protease inhibitors, respectively. However, RAVs to NS5B nucleo(t)ide inhibitor (NI) and NI-based combinations were uncommon (<4% of sequences). As expected, combinations of multiple RAVs to the IFN-free regimens recommended by current guidelines were rarely detected (0.2%–2.0%). Our results showed that the overall global prevalence of DAA RAVs was high irrespective of geography or genotype. However, the NI-based multi-DAA regimens had a low RAV prevalence, suggesting that these regimens are the most promising strategies for cure of the long-term HCV infection.

Hepatitis C virus (HCV) infection is a global health problem, with more than 170 million individuals infected worldwide^1^. Pegylated-interferon (Peg-IFN) and ribavirin (RBV) are standard treatments for HCV infection; however, adverse reactions to these drugs occur in a significant proportion of patients^2^, and a sustained virological response (SVR) is only achieved in approximately 50% of patients with HCV genotype (GT) 1 infections^3^.

Direct-acting antiviral agents (DAAs) have become the new standard of anti-HCV therapy and have shown an extremely high SVR rate^4^. The advantage of DAA based therapy is the ability to directly inhibit specific HCV proteins that are important for HCV replication in hepatocytes, including non-structural(NS)3/4A protease^5^, NS5A protein^6^ and NS5B polymerase^7^. Several novel anti-HCV compounds have recently been investigated. These include: i) the NS3 protease inhibitors Boceprevir, Telaprevir, Paritaprevir and Simeprevir; ii) the NS5A inhibitors Daclatasvir, Ledipasvir and Omitasvir; and iii) the NS5B nucleo(t)ide inhibitor (NI) Sofosbuvir and non-nucleo(t)ide inhibitor (NNI) Dasabuvir. However, due to the low fidelity of HCV polymerase, the high HCV replication rate and the strong selective pressures on the virus, a collection of HCV quasispecies exist within an infected individual before treatment initiation^8^. Furthermore, novel populations that can contain every potential substitution (some of which convey various degrees of resistance to DAAs) are likely created and lost each day^8^. Indeed, drug resistance associated variants (RAVs) have been observed both *in vitro* and in clinical trials^9^,^10^.

Even though a number of studies have reported frequencies for DAA RAVs^11^,^12^,^13^, the global prevalence of DAA RAVs remains unknown. This information could promote and guide the future development of anti-HCV DAA therapies; therefore, this study aimed to investigate the global prevalence of HCV DAA RAVs.

## Results

### Screening of HCV genomic sequences

**Figure 1:**
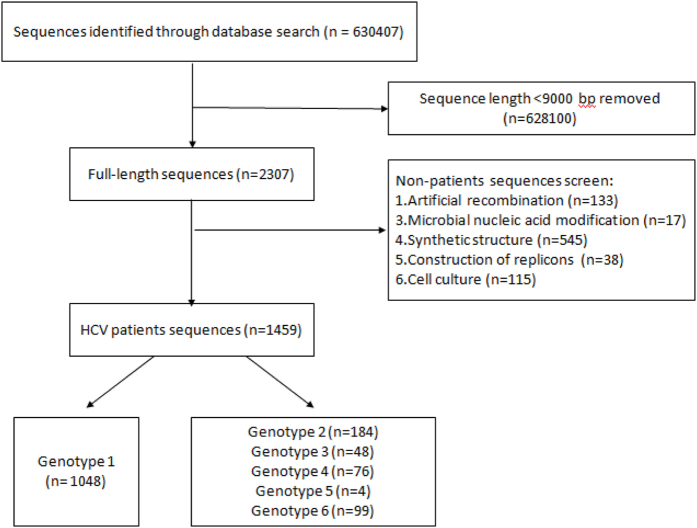
Illustration of GenBank database HCV genome searching and screening strategy.

We identified 630,407 sequences from the NCBI Nucleotide Database in August 2014 using the key words “hepatitis C virus” or “HCV”. After removing sequences with <9000 bp, we narrowed the list of sequences to 2307 sequences of interests. After removing duplicates and non-patient orientated sequences, we obtained a list of 1459 sequences (Fig. 1). Genbank accession numbers for all sequences are provided in Supplementary Table 1. Among these sequences, 91% (1327/1459) were confirmed to be DAA-naïve by searching for their annotated information and retrieving all DAA-related clinical trials since 2003.

To investigate the prevalence of described RAVs in relation to investigational DAAs, we analyzed related amino acid substitutions separately for the 687 GT1a, 361 GT1b, 184 GT2, 48 GT3, 76 GT4 and 99 GT6 HCV sequences. The prevalence of RAVs in GT5 was not assessed because of the small number of available samples (n = 4).

### Identification of DAA RAVs

Most RAVs to examined DAAs were infrequent (0.1%–3.5%, Table 1). However, there were several exceptions for different genotypes. In the NS3 region, the Q80K variant (associated with resistance to Simeprevir) was the most frequently observed among the GT1a sequences (37.6%, 258/687). In contrast, the variant S122T to Simeprevir was the most frequently detected (5.5%, 20/361) in GT1b sequences. The variants L31M, P58S and Y93H in the NS5A region and the variants L159F to Sofosbuvir and S556G to Dasabuvir in NS5B region were common in GT1b sequences (3.8%–9.7%). For other GTs, the variant S122R to Simeprevir in the NS3 region and the variant H58P to Daclatasvir in the NS5A region were common in GT2 sequences (45.1%, 78/173 and 50.8%, 88/173). The Q30K variant to Daclatasvir and Ledipasvir in the NS5A region was observed in 29.2% of GT3 sequences. The Q30R variant to all three NS5A inhibitors was mainly observed in the GT4 and GT6 sequences (55.3% and 24.2%, respectively). Furthermore, the I170V variant to Boceprevir in the NS3 region and the variants M28V and Y93S to at least two NS5A inhibitors in the NS5A region were common in GT6 sequences as well (22.2%–65.7%; Table 1).

### Global prevalence of DAA RAVs

**Figure 2:**
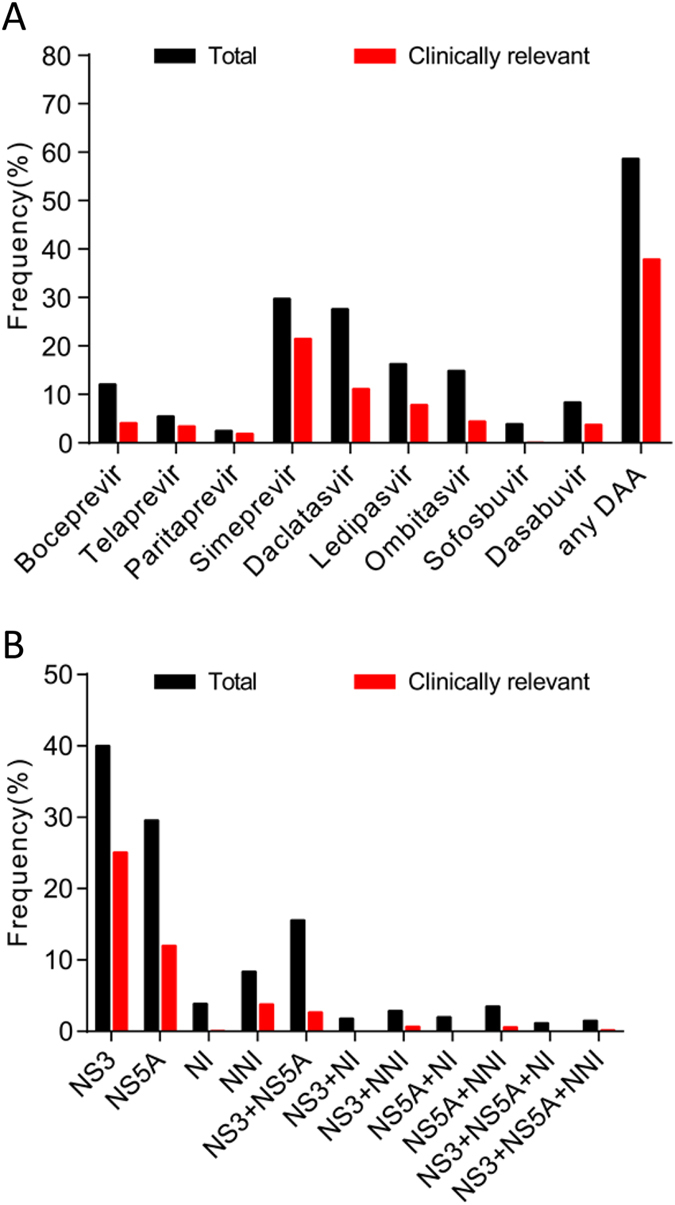
The prevalence of total and clinically relevant DAA resistance associated variants to different DAAs (**A**) and regions (**B**).

**Figure 3:**
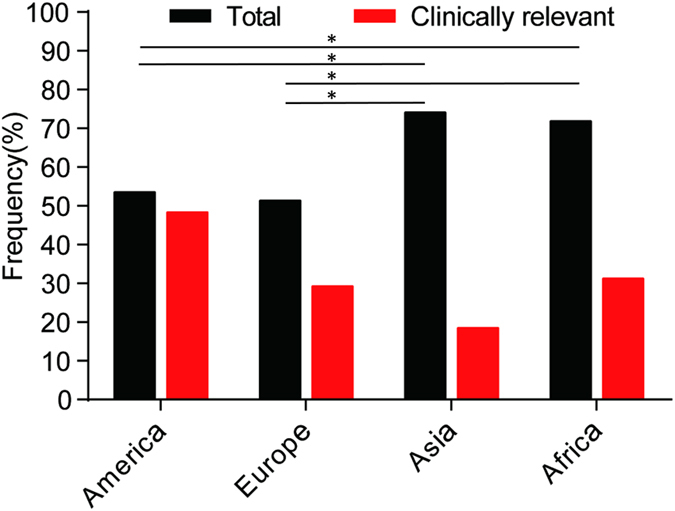
The geographic prevalence of total and clinically relevant DAA resistance associated variants. Oceania was not assessed due to the low number of available samples (four sequences). *p < 0.05.

The overall prevalence of RAVs to all nine DAAs examined was 58.7% (854/1455). When the analysis was more conservatively restricted to clinically relevant RAVs, 37.9% of the total sequences harbored as least one RAV (Fig. 2A). Geographically, the overall prevalence of RAVs in America, Europe, Asia and Africa was 53.5% (433/810), 51.4% (116/227), 74.1% (275/372) and 71.9% (30/42), respectively. The resistance rates observed in Asia and Africa were much higher than those observed in Europe and America (*p* < 0.05). The prevalence of clinically relevant RAVs was 48.4% in America, 29.3% in Europe, 18.5% in Asia and 31.3% in Africa. Oceania was excluded from this analysis because of the limited number of samples (four sequence; Fig. 3).

### Prevalence of RAVs to different DAA regimens

The overall prevalence of RAVs to NS3 protease inhibitors was high and was followed by NS5A inhibitors. The overall prevalence to NS5B polymerase inhibitors was low. RAVs occurring in the NS3 region comprised 40.0% of the total sequences, and were associated with resistance to Boceprevir (12.1%), Telaprevir (5.5%), Simeprevir (29.8%) and Paritaprevir (2.5%). RAVs were harbored in 29.6% of the sequences in the NS5A region and were associated with resistance to Daclatasvir (27.6%), Ledipasvir (16.3%) and Omitasvir (14.9%). Notably, RAVs to NS5B NI Sofosbuvir and NNI Dasabuvir were uncommonly detected (3.9% and 8.4%, respectively; Fig. 2A,B). When clinically relevant RAVs were analyzed, 25.1% of the sequences detected were RAVs in the NS3 region. These RAVs were primarily comprised of RAVs to Simeprevir (21.5%). RAVs to other NS3 inhibitors were uncommon (1.9%–4.1%). The prevalence of RAVs in the NS5A region was 12.0%, and these RAVs were associated with resistance to Daclatasvir (11.2%), Ledipasvir (7.8%) and Omitasvir (4.4%). Only 0.1% and 3.8% of the sequences examined were RAVs to NI Sofosbuvir or NNI Dasabuvir, respectively (Fig. 2A,B).

Furthermore, the overall prevalence of combinations of multiple RAVs in different NS regions of the same sequence was low (1.2%–3.5%; Fig. 2B). One exception to this overall trend was the combination of multiple RAVs in both NS3 and NS5A regions (15.6%). Regarding clinically relevant RAVs, multiple RAVs with the same sequence were infrequent, especially NS5B related combinations. For NS5B, nearly no sequences were detected for the combination of multiple clinically relevant RAVs (0.2%–0.7%; Fig. 2B).

### Prevalence of RAVs in various genotypes

**Figure 4:**
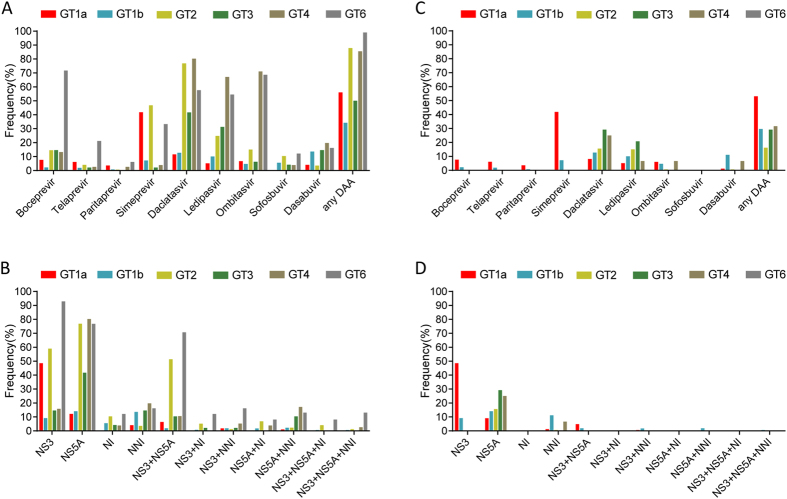
The prevalence of resistance associated variants in various genotypes (GTs). Frequency of all RAVs for different DAAs (**A**) and regions (**B**). Frequency of clinically relevant RAVs for different DAAs (**C**) and regions (**D**).

In GT 1a, the total frequency of RAVs was 56% and the highest prevalence of RAVs was observed in the NS3 region, especially in Simeprevir. In GT 1b, the total frequency of RAVs was 34.3% and the RAVs were mainly detected in the NS5A region, particularly in Daclatasvir. Notably, the prevalence of RAVs in NS5B related combinations was low, irrespective of GT 1a or 1b (Fig. 4A,B). The most commonly observed clinically relevant RAVs were RAVs to Simeprevir in GT1a and Daclatasvir in GT 1b (41.9% and 12.7%, respectively; Fig. 4C).

In other GTs, the overall prevalence of RAVs in GT2, GT3, GT4 and GT6 were 87.9%, 50%, 85.5% and 99%, respectively (Fig. 4A). The highest prevalence of RAVs in these GTs occurred in the NS5A region (41.7%–80.3%). Additionally, the RAVs in the NS3 region in GT2 (mainly observed to Simeprevir) and GT6 (mainly observed to Boceprevir and Simeprevir) were also common (59% and 92.9%, respectively). However, the RAVs to NI Sofosbuvir related combinations were uncommon in GT3 and GT4 (2.1%–3.9%), but frequent in GT2 and GT6 (4.0%–12.1%; Fig. 4A,B). Further analysis of clinically relevant RAVs indicated that 16.2%, 29.2% and 31.6% of the sequences were observed RAVs in GT2, GT3 and GT4, respectively. Clinically relevant RAVs in the NS5A region (mainly observed to Daclatasvir and Ledipasvir) were frequent in these GTs (15.6%–29.2%). Remarkably, none of the sequences observed in these GTs corresponded to multiple clinically relevant RAVs to NI related combinations (Fig. 4C,D).

### Prevalence of RAVs to IFN-free regimens

IFN-free regimens were recently recommended for the clinical treatment of HCV infections by the Asian Pacific Association for the Study of the Liver (APASL)^14^, the European Association for the Study of the Liver (EASL)^15^ and the American Association for the Study of Liver Disease (AASLD)^16^. These recommended regimens included Sofosbuvir plus Ribavirin treatment for GT2 and GT3 patients; Sofosbuvir plus Simeprevir for GT1 and GT4 patients; Sofosbuvir plus Ledipasvir for GT1, GT4, GT5 and GT6 patients; Sofosbuvir plus Daclatasvir for all GTs and the combination of Paritaprevir, Ritonavir or Ombitasvir with Dasabuvir (3D) for GT1 naïve patients.

**Figure 5:**
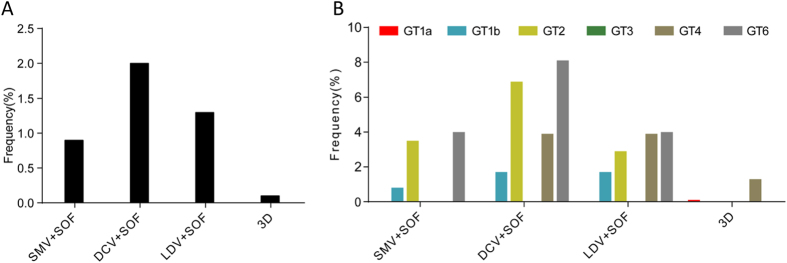
The prevalence of resistance associated variants to IFN-free regimens in total (**A**) **and among various genotypes** (**B**). GT, genotype; SMV, Simeprevir; SOF, Sofosbuvir; DCV, Daclatasvir; LDV, Ledipasvir; 3D, Paritaprevir and Ombitasvir plus Dasabuvir.

Multiple RAV combinations to these IFN-free regimens were observed, but the frequencies were extremely low. Only a few sequences were detected that included the combination of multiple RAVs associated with resistance to Simeprevir plus Sofosbuvir, Daclatasvir plus Sofosbuvir, Ledipasvir plus Sofosbuvir and Paritaprevir/Ombitasvir plus Dasabuvir (0.9%, 2.0%, 1.3% and 0.1%, respectively; Fig. 5A). Similarly, in different GTs, the total prevalence of multiple RAV combinations to these regimens was also low. An exception to these observations was the combination of multiple RAVs to the regimen Sofosbuvir plus Daclatasvir in GT2 and GT6, and this was observed in 6.9% and 8.1% sequences, respectively (Fig. 5B). Remarkably, multiple clinically relevant RAV combinations to these IFN-free regimens were not detected in this study.

## Discussion

Our current study demonstrated that the global prevalence of DAA RAVs was high (58.7%, 854/1455; between 53.5% and 74.1% in various geographical locations or between 48.4% and 99.0% in the HCV genotypes examined). RAVs in the NS5A and NS3 regions were most frequently observed; however, RAVs in the NS5B region were rare, especially in association with the recommended IFN-free regimens (0.1%–2.0%). As with clinically relevant RAVs, the prevalence of RAVs in these regions was lower.

RAVs were detected in up to 58.7% of the sequences analyzed in this study. This frequency is significantly higher than that observed in the previous study by Kuntzen *et al.*^17^ which reported that HCV genome dominant DAA resistance variants occurred in 8.6% of treatment-naïve HCV genotype 1-infected patients in American and European populations. The huge discrepancy between these studies may be the result of several factors. First, the current study included RAVs in the NS3, NS5A and NS5B regions, but Kuntzen *et al.* only included the RAVs in the NS3 and NS5B regions. Second, more GT sequences were enrolled in the current study than the Kuntzen *et al.* study, further contributing to the discrepancy. Finally, the current understanding of HCV DAA RAVs is continuously improving, and more RAVs had been identified at the time of the current study (e.g. the variants at position 80 and 122 in NS3 region) than were available at the time of the Kuntzen *et al.* study. However, Mo *et al.*^18^ reported a prevalence of the RAVs in 80 DAA treated patients with HCV genotype-1 that was significantly higher than that observed in the current study (94% *vs.* 58.7%). One explanation of this discrepancy may be that HCV adapts its genome to survive and increases its resistance to DAA treatment both during and after DAA treatment. Thus, additional variants with increased resistance will occur in DAA-treated patients when compared with DAA-naïve patients^19^.

The current study showed that RAVs to NS5A and NS3 inhibitors were common and occurred with a higher frequency than the frequency reported by previous studies^17^,^20^. This discrepancy might be due to the smaller sample sizes of the previous studies. Furthermore, the body of knowledge concerning DAA RAVs continues to grow, and discrepancies between the current and previous studies may be the result of an increase in the number of known RAVs. The variants L31M and Y93H, which induce resistance to Daclatasvir and asunaprevir, were recently detected by ultra-deep sequencing analysis^21^. These variants were infrequently detected in the current study (1.8% and 4.3%, respectively). Conversely, the Q80K variant associated with Simeprevir resistance in GT1a patients^22^ was more common in the current study (37.6%, 258/687). This result was supported by the results reported in another recent study^18^. The frequency of NS5B inhibitor RAVs was low in this study, especially RAVs to NI. Notably, the S282T variant in the NS5B region leading to Sofosbuvir resistance^23^,^24^ occurred in just one sequence. This observation was consistent with a previous study^20^.

Mono-therapy with NS3 inhibitors resulted in the early emergence of drug resistance variants^25^. Therefore, the use of drug combinations, especially drugs with different mechanisms of action against HCV infection, could lead to a reduction in drug resistance and RAVs. Several clinical trials implementing various DAA combinations have reported increased SVR, lower resistance rates and better drug safety profiles^26^. In this study, RAVs to the different combinations of DAAs were uncommon, especially RAVs to NI-related combinations of DAAs. Furthermore, when compared with the relatively low SVR and serious adverse effects associated with IFN therapy, the IFN-free regimens were a more effective anti-HCV treatment, especially in patients who could not bear IFN or treatment-failure with IFN. Some IFN-free regimens have recently been recommended by EASL, APASL and AASLD and have shown extremely high SVR. Combinations of multiple RAVs in the same sequence to the recommended IFN-free regimens were rare in the present study. This indicates that IFN-free regimens are more effective and should be considered the superior choice for clinical anti-HCV therapy.

The current study is novel and has a number of important strengths. First, we utilized full-length HCV genome sequences to analyze DAA resistance. This included all DAA resistance regions (NS3, NS5A and NS5B region). Second, we included all up-to-date approved DAA data in our data analysis. However, this study has some limitations as well. HCV genome sequence data were obtained from the NCBI nucleotide database. It is possible that some detailed information could be missing from these database entries, so the potential of bias cannot be ruled out. For example, the database contained few Oceanic sequences and GT5 sequences, which hindered further analyses of these sequences sub-populations.

In summary, the global prevalence of DAA RAVs was high, independent of global regions or HCV genotypes. Furthermore, the high frequencies mainly occurred in the NS5A and NS3 regions. However, RAVs to NI-related multiple DAAs were rare, suggesting that NI-based combination therapy is a promising strategy for HCV infection elimination. Our current data supports the EASL, APASL and AASLD recommendations of IFN-free regimens for HCV infection control.

## Methods

### GenBank search strategy

HCV genomic sequences were retrieved from GenBank (http://www.ncbi.nlm.nih.gov/) in August of 2014 using the key words “hepatitis C virus” or “HCV.” After the initial search, near full-length HCV sequences (>9000 bp) were screened and any duplicate sequences or sequences from non-human hosts were discarded (Fig. 1). Finally, the following information was extracted for each sequence: GenBank Accession Number, serum or plasma collection time and geographic data.

### HCV genotypes

HCV genotypes were retrieved and identified with the NCBI viral genotyping tool (http://www.ncbi.nlm.nih.gov/projects/genotyping/formpage.cgi).

### Variant analyses and definition

**Figure 6:**
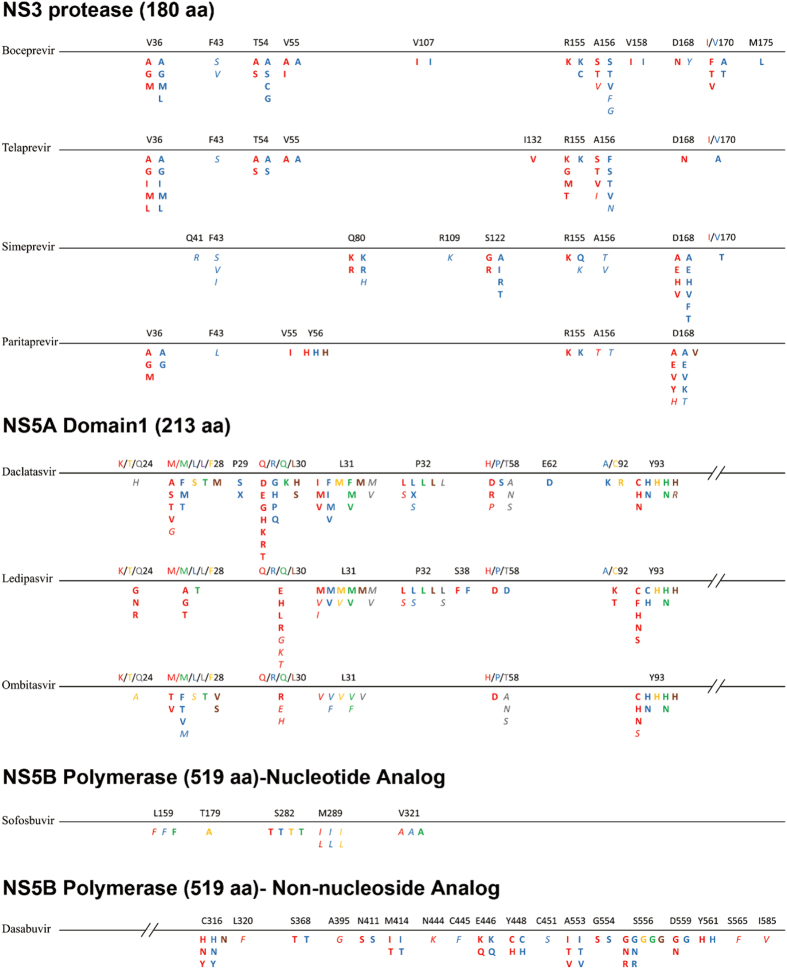
Resistance associated variants for NS3 protease inhibitors, NS5A inhibitors and NS5B polymerase inhibitors. Variants are color-coded based on genotype: 1a, red; 1b, blue; 2, yellow; 3, green; 4, brown; 6, grey. Clinically relevant RAVs are highlighted in bold, *in vitro* RAVs are identified by italics. Note: X, amino acid deletion.

All DAA RAVs included in this study were identified from the most current available literature, as summarized in Fig. 6 27,28,29,30,31,32,33,34,35,36,37,38,39,40,41,42,43,44,45,46,47. To facilitate investigation of the prevalence of RAVs, clinically relevant RAVs selected during or after drug treatment in patients and obtained in phenotypic assays were differentiated from drug resistance variants observed *in vitro*. Little data has been published concerning RAVs for GT2–GT6, thus the information available concerning RAVs for these GTs was limited. Therefore, when information concerning clinically relevant RAVs for GT2–GT6 was missing, RAVs in GT1 were used as substitute *in vitro* RAVs for GT2–GT6. Sequences were aligned and analyzed with MEGA 5.0 software (Center for Evolutionary Medicine and Informatics, Tempe, AZ, USA). A variant type was described as the replacement of the consensus amino acid in the corresponding genotype with a novel one; for instance, Y93H and Y93N in the NS5A region were described as two variant types.

### Statistical analyses

All data were presented as rates (%) and analyzed statistically using the chi-squared test with SPSS 17 software (SPSS Inc., Chicago, IL, USA). *p* values were calculated with two-tailed statistical analysis, and a *p* value ≤ 0.05 was considered statistically significant.

## Additional Information

**How to cite this article**: Chen, Z.-W. *et al.* Global prevalence of pre-existing HCV variants resistant to direct-acting antiviral agents (DAAs): mining the GenBank HCV genome data. *Sci. Rep.* **6**, 20310; doi: 10.1038/srep20310 (2016).

## Supplementary Material

Supplementary Information

## Figures and Tables

**Table 1:** RAVs detected for NS3, NS5A and NS5B inhibitors in various genotypes.

DAA class	Drug used	RAVs	GT1a n = 687	GT1b n = 361	GT2 n = 184	GT3 n = 48	GT4 n = 76	GT6 n = 99	DAA class	Drug used	RAVs	GT1a n = 687	GT1b n = 361	GT2 n = 184	GT3 n = 48	GT4 n = 76	GT6 n = 99
NS3	BOC	V36A	1						NS5A	DCV	P58S		14	4	1		7
		V36G	1								E62D		1	1	2		5
		V36M	2		1			1			Y93H	4	17	1		5	
		V36L						12			Y93C	5					
		T54A			1						Y93N	1					
		T54S	12	6	1	1	2				Y93R					1	
		T54G					1			LDV	K24G				2		
		V55A	8								K24R	7					3
		V55I	13								M28A						3
		V107I	13								M28G						1
		R155K	6								M28T	3					2
		A156V						1			Q30G	2					
		V158I			3	1	2	3			Q30H	7					1
		I170V	22		18	5	1	65			Q30L	1			2		
		M175L		2	1						Q30R	1		18	1	42	24
	TVR	V36A	1								Q30K				14		
		V36G	1								Q30T				2	4	2
		V36I		1				7			L31I						2
		V36M	2		1			1			L31M	14	14	27	8		
		V36L	11					12			L31V	1	3		2		
		T54A			1						H58D	2					
		T54S	15	6	1	1	2				A92T	1	5			1	
		V55A	9								Y93C	5					
		I132V	5		4	1	2				Y93H	4	17	1		5	
		R155K	6								Y93N	1					
		A156V						1			Y93S			5		3	22
	SMV	Q80K	258	2	1			19		OMV	T24A			1			
		Q80R	4	1		1					M28T	3					2
		S122G	31				1				M28V	26					25
		S122T		20	2			9			L28M					11	4
		S122R			78						L28T			2			
		R155K	6								F28S			1			
		A156V						1			Q30H	8					1
		D168T					1				Q30R	1		18	1	42	24
		D168E	2	3			1	5			L31V	1	3		2		
		D168V	1								H58D	2					
	PTV	V36A	1								Y93C	5					
		V36G	1								Y93H	4	17	1		5	
		V36M	2		1			1			Y93N	1					
		V55I	13								Y93S			5		3	22
		R155K	6						NS5B	SOF	L159F		19				2
		D168E	2	3			1	5			T179A			9			
		D168T					1				S282T		1				
		D168V	1								M289I			7			
NS5A	DCV	M28A						1			M289L			5	2	3	11
		M28V	26					25		DSV	C316H					3	
		M28T	3					2			C316Y		1				
		M28G						1			C316N	1				5	
		L28M					11	4			N411S				1		
		L28T			2						M414I		1			4	
		F28S			1						C445F		4				
		Q30G	2								E446Q				3		
		Q30H	8					1			Y448C		1				
		Q30R	1		18	1	42	24			Y448H	2					
		Q30T				2	4	2			C451S		4				
		Q30K				14					A553V		1				4
		L30S					6				G554S			1	2		
		L31M	14	14	27	8					S556G	4	35	1			1
		L31V	1	3		2					S556N	1			5		
		L31I		2				2			S556R		1				14
		H58D	2								D559G						1
		H58R	1				1				I585V	20	5	1	3	1	4
		H58P	27		88		17										

GT, genotype; BOC, Boceprevir; TVR, Telaprevir; SMV, Simeprevir; PTV, Paritaprevir; DCV, Daclatasvir; LDV, Ledipasvir; OMV, Ombitasvir; SOF, Sofosbuvir; DSV, Dasabuvir.
